# Investigating the role of pentraxin 3 as a biomarker for bacterial infection in subjects with COPD

**DOI:** 10.2147/COPD.S123528

**Published:** 2017-04-18

**Authors:** Samantha J Thulborn, Madiha Dilpazir, Koirobi Haldar, Vijay Mistry, Christopher E Brightling, Michael R Barer, Mona Bafadhel

**Affiliations:** 1 Respiratory Medicine Unit, Nuffield Department of Medicine, University of Oxford; 2 Department of Biological and Medical Sciences, Oxford Brookes University, Oxford; 3 Department of Immunity, Infection & Inflammation, University of Leicester, Leicester, UK

**Keywords:** TNF-inducible gene 14 protein, infection, sputum

## Abstract

**Background:**

Pentraxin 3 (PTX3) is an acute phase protein, involved in antibacterial resistance. Recent studies have shown PTX3 levels to be elevated in the presence of a bacterial infection and in a murine sepsis model.

**Objective:**

We aim to investigate if sputum PTX3 can be used as a biomarker for bacterial infection in subjects with COPD.

**Materials and methods:**

Sputum samples from 142 COPD patients (102 men) with a mean (range) age of 69 years (45–85) and mean (SD) post-bronchodilator percentage predicted forced expiratory volume in 1 second (FEV_1_) of 50% (19) were analyzed for PTX3, using a commercial assay at stable state and during an exacerbation. Association with bacteria, from culture, quantitative real-time polymerase chain reaction (qPCR) and colony-forming units (CFU) was investigated.

**Results:**

The geometric mean (95% CI) PTX3 level at stable state was 50.5 ng/mL (41.4–61.7). PTX3 levels correlated with absolute neutrophil count in sputum (*r*=0.37; *P*<0.01), but not FEV_1_ or health status. There was a weak correlation between PTX3 and bacterial load (CFU: *r*=0.29, *P*<0.01; 16S qPCR: *r*=0.18, *P*=0.05). PTX3 was a poor predictor of bacterial colonization (defined as >10^5^ CFU/mL at stable state) with a receiver-operating characteristic (ROC) area under the curve (AUC) of 0.59 and 95% confidence interval (CI) 0.43–0.76 (*P*=0.21). During an exacerbation, there was a modest increase in PTX3 (fold difference 0.15, 95% of difference 0.02–0.29; *P*=0.02), and PTX3 fared better at identifying a bacteria-associated exacerbation (ROC AUC 0.65, 95% CI 0.52–0.78, *P*=0.03).

**Conclusion:**

PTX3 is associated with bacterial infection in patients with COPD, but its utility as a biomarker for identifying a bacteria-associated exacerbation warrants further studies.

## Introduction

COPD is characterized by persistent airflow limitation, which is progressive and associated with an enhanced inflammatory response to noxious agents.^1^ Exacerbations of COPD have a significant effect on the health of patients^2^ and are associated with microbial^3^–^7^ and airway inflammation.^8^ Chronic bacterial infection, defined as >10^5^ colony-forming units (CFU),^9^ occurs in 30% of patients at stable state^10^ and increases up to 50% during an exacerbation.^3^ Guidelines for treatment of an exacerbation advocate the use of systemic corticosteroids^11^ and antibiotics;^12^ however, this is usually without evidence of a bacterial infection as standard culture techniques are not rapid.^13^ The occurrence of resistant bacteria is a growing concern^14^ and deemed an emerging medical catastrophe.^15^ The rapid identification of a bacterial exacerbation is urgently needed to aid clinical treatment decisions. One such possible marker is pentraxin 3 (PTX3). PTX3 is a soluble pattern recognition receptor,^16^ recognizing pathogen-associated molecular patterns expressed by microorganisms.^17^ PTX3 is induced in response to proinflammatory stimuli^16^ and toll-like receptor (TLR) interactions^17^ and contributes to innate resistance to pathogens.^16^ PTX3 levels have been shown to be elevated in a murine bacterial infection model,^18^ in sepsis,^19^–^21^ and in inflammatory rheumatic disease.^22^ In this study, we aim to determine whether sputum PTX3 is a sensitive biomarker for bacterial colonization at stable state and for a bacterial exacerbation in patients with COPD.

## Materials and methods

### Subjects and sampling

COPD subjects entering a longitudinal study looking at biomarkers in COPD were analyzed, where subject inclusion and exclusion criteria, study design, and measurements are as previously described.^3^ In brief, subjects attended a stable state visit every 3 months over a 12-month period and also during exacerbations and 2 weeks post-exacerbation. An exacerbation event was defined according to Anthonisen criteria^23^ and NICE guidance,^24^ and a bacteria-associated exacerbation was defined as >10^7^ CFU/mL as previously described.^3^ At each visit, participants underwent pre- and postbronchodilator spirometry; blood was collected by standard venipuncture and sputum induction for sputum collection. If participants were unable to perform sputum induction, spontaneous sputum was collected. Health status and symptoms were measured using the St George’s respiratory questionnaire (SGRQ),^25^ MRC dyspnea scale,^26^ the chronic respiratory disease questionnaire (CRQ),^27^ and the visual analog score (VAS).^28^ Chronic bacterial infection (colonization) was defined as >10^5^ CFU/mL at stable state^9^ and in this cohort was associated with a high positive predictive value of a respiratory pathogenic microorganism (Table 1). All subjects gave written informed consent, and the study was approved by the Leicestershire, Northamptonshire and Rutland ethics committee (reference number: 07/H0406/157).

### Sputum processing

Sputum processing involved plug selection, followed by a dispersion step with Dulbecco phosphate-buffered saline and a mucolytic step with dithiothreitol as previously described.^3^ A filtration step to remove debris for cytospin preparation and quantification of cell differential count was then performed.^29^ Samples with a cell viability of <40% and a squamous contamination >20% were excluded. CFUs were prepared for semi-quantitative analysis using standard techniques.^30^ A further 500 μL of the filtrate was processed by SYBR green (Applied Biosystems^®^; Life Technologies Corp., Carlsbad, CA, USA) quantitative polymerase chain reaction (qPCR) for *Haemophilus influenzae* and *Staphylococcus aureus* bacterial DNA, and a Taqman qPCR assay for the quantification of *Streptococcus pneumoniae* and *Moraxella catarrhalis* as described previously.^3^ Viral RNA was extracted, using in-house assays, from an additional sputum plug for PCR analysis, as previously described.^3^ Only samples with available sputum microbiology were analyzed. A sputum eosinophilia was defined as a sputum eosinophil count of >3%.^31^

### Pentraxin 3 Quantikine ELISA

A Human Pentraxin 3/TSG-14 Quantikine ELISA Kit (R&D Systems, Oxfordshire, UK) was used to measure PTX3 levels; the assay was carried out as per manufacturer’s protocol and read using an EnVision plate reader (Perkin-Elmer, MA, USA). The lower limit of detection for PTX3 is 0.31 ng/mL. Readings taken at 560 nm wavelengths were subtracted from the readings taken at 450 nm to correct for any optical imperfections within the plate. The average optical density from the negative control was subtracted from each well to remove any background noise present. All standards, negatives, and samples were run in duplicate on each plate. The standards were plotted against the optical density value. The *R*^2^ value was >0.97.

### Statistical analysis

GraphPad Prism version 6 (GraphPad Software, Inc., La Jolla, CA, USA) and Statistical Package for the Social Sciences (SPSS) Statistics version 22 (SPSS, Inc., Chicago, IL, USA) were used for statistical analysis. The Kolmogorov–Smirnov test was applied for normality. PTX3 levels were log-normal transformed. All parametric data were displayed as mean and standard deviation (SD), all non-parametric data were displayed as median (interquartile range [IQR]), and log-transformed data were presented as geometric mean and 95% confidence interval. Paired and unpaired *t*-test and one-way analysis of variance (ANOVA) tests were used to compare two, three, or more groups, respectively. Receiver-operating characteristic (ROC) curves were utilized to measure the sensitivity and specificity of PTX3 as a biomarker. Repeatability analysis was conducted in 10 subjects over three stable visits and the coefficient of variation was used to test repeatability over time. For multiple exacerbation visits, only the first exacerbation was analyzed. A probability of *P*<0.05 was considered to be statistically significant.

## Results

PTX3 was measured at stable state in 148 individual subjects, 6 of these were unreadable and so excluded from analysis; thus, a total of 142 subject samples were analyzed. A paired exacerbation result was available in 95 subjects. The baseline characteristics of the COPD subjects are presented in Table 2.

### Stable state

**Figure 1:**
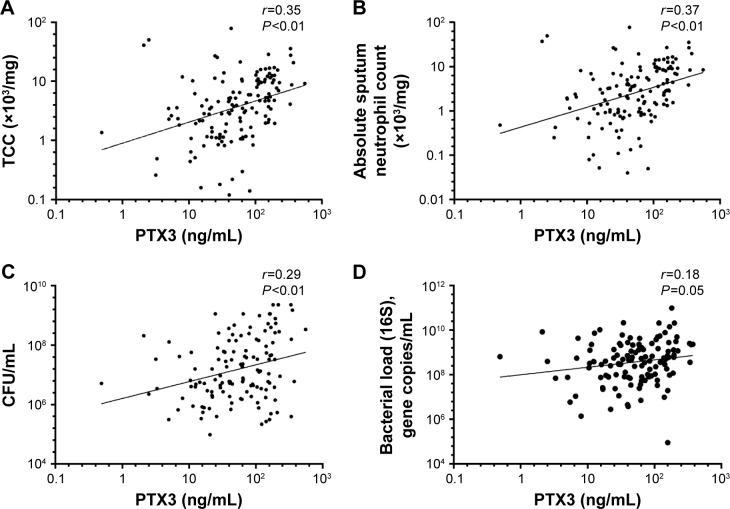
Correlations between PTX3 and (**A**) TCC in sputum; (**B**) absolute neutrophil count in sputum; (**C**) CFU/mL (**C**); and (**D**) bacterial load measured by 16S quantitative polymerase chain reaction. **Abbreviations:** CFU, colony forming units; PTX3, pentraxin 3; TCC, total cell count.

**Figure 2:**
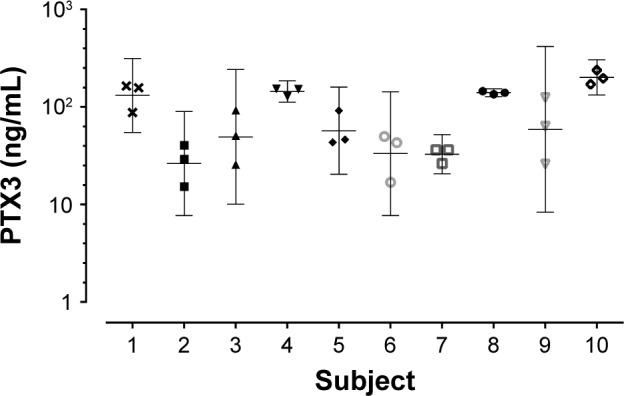
PTX3 levels from 10 subjects over three stable visits. **Note:** Mean 95% CI. **Abbreviations:** CI, confidence interval; PTX3, pentraxin 3.

The geometric mean (95% CI) of PTX3 was 50.5 ng/mL (41.4–61.7). Ex-smokers compared to current smokers had higher PTX3 levels (geometric mean [95% CI]: ex-smokers 61.3 ng/mL [48.5–77.4] vs current smokers 40.6 [28.3–58.3], *P*=0.05). PTX3 was not associated with severity of COPD, defined by Global Initiative for Chronic Obstructive Lung Disease (GOLD) (2007)^32^ or treatment with or without inhaled corticosteroids (ICS). There were no correlations seen between PTX3 and the post-bronchodilator forced expiratory volume in 1 second (FEV_1_) (*r*=−0.13; *P*=0.15), subject health (MRC: *r*=0.04, *P*=0.66; CRQ: *r*=−0.07, *P*=0.40; SGRQ: *r*=0.05, *P*=0.54; and VAS: *r*=−0.01, *P*=0.91), CRP (*r*=0.13, *P*=0.14), or absolute blood neutrophils (*r*=0.09, *P*=0.27). PTX3 was associated with sputum total cell count (*r*=0.35, *P*<0.01; Figure 1A) and neutrophilic airway inflammation (*r*=0.37, *P*<0.01; Figure 1B). PTX3 was associated with bacterial load, measured by CFU/mL (*r*=0.29, *P*<0.01; Figure 1C) and total 16S qPCR (*r*=0.18, *P*=0.05; Figure 1D). Non-eosinophilic subjects had significantly higher PTX3 levels than eosinophilic subjects (fold difference [95% CI] 0.30 [0.10–0.50]; *P*<0.01). For individual pathogens, pathogen-specific qPCR demonstrated a weak but non-significant correlation of PTX3 with *S. pneumoniae* (*r*=0.33; *P*=0.38) but not *M. catarrhalis* (*r*=0.01; *P*=0.98) or *H. influenzae* (*r*=−0.04; *P*=0.84). There were too few single *S. aureus* qPCR isolates to analyze. PTX3 was not a predictor of bacterial colonization at stable state (ROC area under the curve [AUC] 0.59, 95% CI 0.43–0.76, *P*=0.21) and was stable over time with a coefficient of variation 10% (Figure 2).

### At exacerbation

**Figure 3:**
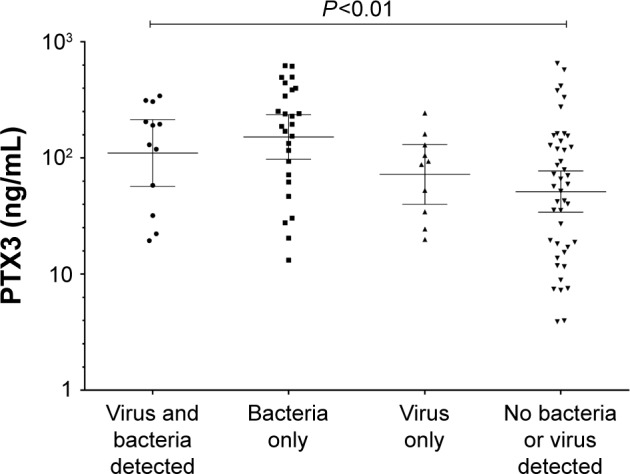
PTX3 levels when divided according to the presence of a virus or bacterial infection. **Note:** Mean 95% CI. **Abbreviations:** CI, confidence interval; PTX3, pentraxin 3.

**Figure 4:**
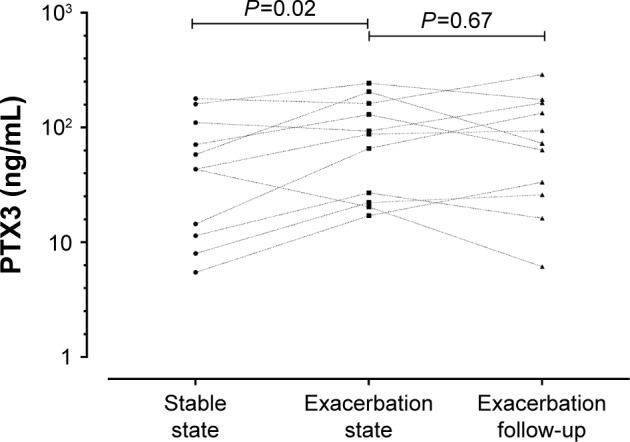
PTX3 levels in 11 paired sputum samples at stable, exacerbation, and 2 weeks post-exacerbation treatment. **Abbreviation:** PTX3, pentraxin 3.

During an exacerbation, there was a significant increase in PTX3 levels from a stable to exacerbation state (fold difference 0.15, 95% of difference 0.02–0.29; *P*=0.02). Subjects with an increase in PTX3 at the exacerbation were also more likely to culture a pathogen (Table 3). PTX3 at exacerbation correlated with CFU/mL (*P*<0.01, *r*=0.30) but not total 16S (*P*=0.91, *r*=−0.01). At exacerbation, there was a moderate but non-significant correlation of PTX3 with *H. influenzae* single isolate pathogen-specific qPCR (*r*=0.41, *P*=0.18) but not *M. catarrhalis* (*r*=−0.12, *P*=0.82). There were too few isolates of *S. pneumoniae* and *S. aureus* to analyze. A PTX3 level of 118.0 ng/mL had a sensitivity and specificity of 60%, respectively (ROC AUC 0.65, 95% CI 0.52–0.78, *P*=0.03) to determine a bacterial exacerbation. The presence or absence of viruses at the onset of an exacerbation did not affect PTX3 levels (fold difference [95% CI] of 0.03 [−0.25 to 0.32]; *P*=0.81). The combination of bacteria and virus detection at exacerbation demonstrated similar PTX3 levels to bacteria alone (Figure 3). In a small subset of subjects (n=11) with sputum PTX3 measured at a stable, exacerbation and exacerbation follow-up visit, a trend to increase in PTX3 from stable to exacerbation state was seen in the 11 paired subjects and a trend to reduction at follow-up (Figure 4).

## Discussion

In this study, we aimed to determine if PTX3 was a sensitive biomarker for bacterial colonization at stable state and for a bacterial exacerbation in subjects with COPD, by measuring PTX3 concentrations in sputum. We have demonstrated for the first time that PTX3 in subjects with COPD correlates with bacterial burden, both at stable state and exacerbations and increases during an exacerbation event. PTX3 did not correlate with symptoms or lung function and was a reasonable predictor of a bacteria-associated exacerbation.

Previous studies in non-COPD cohorts have shown PTX3 to rapidly increase due to an infection.^19^,^33^ PTX3 has been shown to be elevated in COPD subjects compared with healthy controls in plasma^34^ and sputum.^35^ PTX3 levels have been correlated with the number of neutrophils present in sputum and have been shown to be elevated in current and ex-smokers compared with non-smokers.^36^ Our results support these statements. To our knowledge, no study has looked into PTX3 as a viable biomarker for a bacterial colonization or bacterial exacerbation in COPD subjects, though this has been suggested.^37^

PTX3 levels have previously been linked to inflammatory diseases;^22^ so it was not surprising to see a correlation between PTX3 levels and neutrophils in sputum, a pro-inflammatory cell,^38^ and also a cell involved in PTX3 storage.^17^ PTX3 has also been implemented in bacterial infection,^18^–^21^ and our study supports this as we found a correlation between bacterial load (CFU/mL) in sputum at stable and exacerbation states implying that PTX3 expression in the lungs is driven by bacterial infection. This was further highlighted when PTX3 expression was relatively unchanged with the presence of a virus. Although non-significant, we did identify a moderate correlation of *H. influenzae* individual pathogen-specific qPCR with PTX3 during an exacerbation but not at stable state. This is unsurprising as *H. influenzae* is a commonly isolated pathogen in COPD patients^39^ and is the main driver of inflammation.^40^ Further work is needed to fully investigate the role of individual pathogens in PTX3 expression.

## Limitations

This study has a few limitations. First, we did not have data on PTX3 in healthy controls, which would support whether levels detected in the COPD population are elevated; an additional infection control, such as bronchiectasis, would provide added value. However, this is the first study to look at infection (chronic and thus colonization) versus acute (bacteria-associated) exacerbations demonstrating modest utility of PTX3. Finally, advances in qPCR mean it is now possible to measure bacteria more accurately using plasmids^41^ and to exclude dead bacteria.^42^ As this was not available at the time of this work, we cannot confirm if the modest associations seen are a consequence of the inclusion of live and dead bacteria, and both these advancements in the field would greatly enhance this study.

## Conclusion

To conclude, this study found that PTX3 correlates with bacterial load and that PTX3 is not sensitive or specific enough to be used as a biomarker for bacterial colonization but could be a potential biomarker for a bacteria-associated exacerbation in patients with COPD. However, further work is warranted to fully evaluate this.

## Figures and Tables

**Table 1:** Proportion of subjects below and above the cutoff of 10^5^ CFU/mL that had a negative or positive microbiology results at stable state

Culture results	<10^5^ (n=8)	>10^5^ (n=65)
No significant growth	5	34
Positive pathogen culture (*Haemophilus influenzae, Streptococcus pneumoniae, Moraxella catarrhalis, Staphylococcus aureus, Pseudomonas aeruginosa*)	3	31

**Abbreviation:** CFU, colony-forming units.

**Table 2:** Baseline characteristics of the 142 COPD subjects included in this analysis of pentraxin 3 levels

Subjects, n	142
Male (%)	102 (72)
Age (years)^a^	69 (45–88)
Smokers, n (%)	49 (34)
Ex-smokers, n (%)	88 (62)
Pack year history (units)^a^	52 (10–159)
GOLD 1 (%)	4 (3)
GOLD 2 (%)	56 (39)
GOLD 3 (%)	50 (35)
GOLD 4 (%)	32 (23)
Post-bronchodilator FEV_1_ (L)	1.30 (0.51)
Post-bronchodilator FEV_1_ (%)	50 (19)
FEV_1_/FVC ratio (%)	25 (25)
SGRQ, total score (units)	53.86 (17.59)
MRC (units)^c^	3.00 (2.00–4.00)
CRQ (units)	4.05 (1.14)
VAS total score (mm)	154.8 (77.56)
Inhaled corticosteroids treatment (%)	128 (90)
Long-acting beta agonist treatment (%)	118 (83)
Sputum neutrophils (%)	71 (22)
Total sputum neutrophil count (×10^3^/mg)^b^	2.50 (1.92–3.25)
Sputum eosinophils (%)^b^	1.16 (0.92–1.47)
Total sputum eosinophil count (×10^3^/mg)^b^	0.15 (0.10–0.19)
Colony-forming units/mL^b^ (×10^7^)	1.12 (0.72–1.74)
Bacterial load in sputum plug^b^ (×10^8^)	2.64 (1.76–3.91)

**Notes:** Unless indicated, all data are presented as mean and standard deviation in brackets. GOLD, Global Initiative for Chronic Obstructive Lung Disease individuals grouped (1–4) by severity of disease; SGRQ, scores ranging from 0 to 100 with higher score indicating worse health status (total score on domains of impact, symptoms, and activity); CRQ, scores range between 1 and 7 with higher score representing better health quality; VAS, performed on 100 mm line from “no symptoms” to “worst symptoms”, higher scores represent worse symptoms (total score addition of measured domains: cough, dyspnea, sputum production, and sputum purulence).

a Mean (range).

b Geometric mean and 95% confidence intervals.

c Median (interquartile range).

**Abbreviations:** CRQ, chronic respiratory disease questionnaire; FEV_1_, forced expiratory volume in 1 second; FVC, forced vital capacity; MRC, medical research council; SGRQ, St George’s respiratory questionnaire; VAS, visual analog scale.

**Table 3:** Difference in culture growth between subjects that saw an increase in PTX3 levels compared with those that saw a decrease from stable to exacerbation state

Microbiology result	Increase in PTX3 (n=57)	No increase (n=38)
*Haemophilus influenzae*	16 (28)	5 (13)
*Moraxella catarrhalis*	5 (8)	1 (3)
*Streptococcus pneumoniae*	7 (12)	1 (3)
*Staphylococcus aureus*	0 (0)	0 (0)
*Pseudomonas aeruginosa*	1 (2)	2 (6)
No significant growth	28 (49)	29 (76)

**Note:** Data presented as n (%).

**Abbreviation:** PTX3, pentraxin 3.

## References

[1] Vestbo J, Hurd SS, Agustí AG (2013). Global strategy for the diagnosis, management, and prevention of chronic obstructive pulmonary disease. Am J Respir Crit Care Med.

[2] Garvey C (2016). Recent updates in chronic obstructive pulmonary disease. Postgrad Med.

[3] Bafadhel M, McKenna S, Terry S (2011). Acute exacerbations of chronic obstructive pulmonary disease: identification of biologic clusters and their biomarkers. Am J Respir Crit Care Med.

[4] Monsó E, Ruiz J, Rosell A (1995). Bacterial infection in chronic obstructive pulmonary disease. A study of stable and exacerbated outpatients using the protected specimen brush. Am J Respir Crit Care Med.

[5] Banerjee D, Khair OA, Honeybourne D (2004). Impact of sputum bacteria on airway inflammation and health status in clinical stable COPD. Eur Respir J.

[6] Sethi S, Murphy TF (2008). Infection in the pathogenesis and course of chronic obstructive pulmonary disease. N Engl J Med.

[7] Papi A, Bellettato C, Braccioni F (2006). Infections and airway inflammation in chronic obstructive pulmonary disease severe exacerbations. Am J Respir Crit Care Med.

[8] Bhowmik A, Seemungal T, Sapsford R, Wedzicha J (2000). Relation of sputum inflammatory markers to symptoms and lung function changes in COPD exacerbations. Thorax.

[9] Miravitlles M, Marín A, Monsó E (2010). Colour of sputum is a marker for bacterial colonisation in chronic obstructive pulmonary disease. Respir Res.

[10] Rosell A, Monsó E, Soler N (2005). Microbiologic determinants of exacerbation in chronic obstructive pulmonary disease. Arch Int Med.

[11] National Clinical Guideline Centre (2010). Chronic Obstructive Pulmonary Disease: Management of Chronic Obstructive Pulmonary Disease in Adults in Primary and Secondary care.

[12] Global Strategy for diagnosis m, and prevention of COPD.

[13] Recommendations of the Clinical Subcommittee of the Medical/Scientific Advisory Committee of the Canadian Cystic Fibrosis Foundation (1993). Microbiological processing of respiratory specimens from patients with cystic fibrosis. Can J Infect Dis.

[14] Erkan L, Uzun O, Findik S, Katar D, Sanic A, Atici AG (2008). Role of bacteria in acute exacerbations of chronic obstructive pulmonary disease. Int J Chron Obstruct Pulmon Dis.

[15] Berkowitz FE (1995). Antibiotic resistance in bacteria. South Med J.

[16] Balhara J, Koussih L, Zhang J, Gounni AS (2013). Pentraxin 3: an immuno-regulator in the lungs. Front Immunol.

[17] Kunes P, Holubcova Z, Kolackova M, Krejsek J (2012). Pentraxin 3(PTX 3): an endogenous modulator of the inflammatory response. Mediators Inflamm.

[18] Soares AC, Souza DG, Pinho V (2006). Dual function of the long pentraxin PTX3 in resistance against pulmonary infection with *Klebsiella pneumoniae* in transgenic mice. Microbes Infect.

[19] Vänskä M, Koivula I, Hämäläinen S (2011). High pentraxin 3 level predicts septic shock and bacteremia at the onset of febrile neutropenia after intensive chemotherapy of hematologic patients. Haematologica.

[20] Bastrup-Birk S, Skjoedt MO, Munthe-Fog L, Strom JJ, Ma YJ, Garred P (2013). Pentraxin-3 serum levels are associated with disease severity and mortality in patients with systemic inflammatory response syndrome. PLoS One.

[21] Huttunen R, Hurme M, Aittoniemi J (2011). High plasma level of long pentraxin 3 (PTX3) is associated with fatal disease in bacteremic patients: a prospective cohort study. PLoS One.

[22] Hollan I, Bottazzi B, Cuccovillo I (2010). Increased levels of serum pentraxin 3, a novel cardiovascular biomarker, in patients with inflammatory rheumatic disease. Arthritis Care Res (Hoboken).

[23] Anthonisen NR, Manfreda J, Warren CPW, Hershfield ES, Harding GKM, Nelson NA (1987). Antibiotic therapy in exacerbations of chronic obstructive pulmonary disease. Ann Int Med.

[24] (2016). Chronic obstructive pulmonary disease.

[25] Weatherall M, Marsh S, Shirtcliffe P, Williams M, Travers J, Beasley R (2009). Quality of life measured by the St George’s respiratory questionnaire and spirometry. Eur Respir J.

[26] MRC Medical Research Council Dyspnoea scale/Breathlessness scale [webpage on the Internet].

[27] Chauvin A, Rupley L, Meyers K, Johnson K, Eason J (2008). Outcomes in cardiopulmonary physical therapy: chronic respiratory disease questionnaire (CRQ). Cardiopulm Phys Ther J.

[28] Brightling CE, Monterio W, Green RH (2001). Induced sputum and other outcome measures in chronic obstructive pulmonary disease: safety and repeatability. Respir Med.

[29] Pizzichini E, Pizzichini MM, Efthimiadis A, Hargreave FE, Dolovich J (1996). Measurement of inflammatory indices in induced sputum: effects of selection of sputum to minimize salivary contamination. Eur Respir J.

[30] Pye A, Stockley RA, Hill SL (1995). Simple method for quantifying viable bacterial numbers in sputum. J Clin Pathol.

[31] Wagener AH, de Nijs SB, Lutter R (2015). External validation of blood eosinophils, FENO and serum periostin as surrogates for sputum eosinophils in asthma. Thorax.

[32] Rabe KF, Hurd S, Anzueto A (2007). Global strategy for the diagnosis, management, and prevention of chronic obstructive pulmonary disease. Am J Respir Crit Care Med.

[33] Bottazzi B, Doni A, Garlanda C, Mantovani A (2010). An integrated view of humoral innate immunity: pentraxins as a paradigm. Ann Rev Immunol.

[34] Kurt OK, Tosun M, Kurt EB, Talay F (2015). Pentraxin 3 as a novel biomarker of inflammation in chronic obstructive pulmonary disease. Inflammation.

[35] Schwingel FL, Pizzichini E, Kleveston T (2015). Pentraxin 3 sputum levels differ in patients with chronic obstructive pulmonary disease vs asthma. Ann Allergy Asthma Immunol.

[36] Van Pottelberge GR, Bracke KR, Pauwels NS, Vermassen FE, Joos GF, Brusselle GG (2012). COPD is associated with reduced pulmonary interstitial expression of pentraxin-3. Eur Respir J.

[37] Liu S, Qu X, Liu F, Wang C (2014). Pentraxin 3 as a prognostic biomarker in patients with systemic inflammation or infection. Mediators Inflamm.

[38] Mantovani A, Cassatella MA, Costantini C, Jaillon S (2011). Neutrophils in the activation and regulation of innate and adaptive immunity. Nat Rev Immunol.

[39] Bafadhel M, Haldar K, Barker B (2015). Airway bacteria measured by quantitative polymerase chain reaction and culture in patients with stable COPD: relationship with neutrophilic airway inflammation, exacerbation frequency, and lung function. Int J Chron Obstruct Pulmon Dis.

[40] Barker BL, Haldar K, Patel H (2014). Association between pathogens detected using quantitative polymerase chain reaction with airway inflammation in COPD at stable state and exacerbations. Chest.

[41] Klein D (2002). Quantification using real-time PCR technology: applications and limitations. Trends Mol Med.

[42] Nocker A, Sossa-Fernandez P, Burr MD, Camper AK (2007). Use of propidium monoazide for live/dead distinction in microbial ecology. Appl Environl Microbiol.

